# Model-interpreted outcomes of artificial neural networks classifying immune biomarkers associated with severe infections in ICU

**DOI:** 10.3389/fimmu.2023.1137850

**Published:** 2023-03-09

**Authors:** Gustavo Sganzerla Martinez, Ali Toloue Ostadgavahi, Abdullah Mahmud Al-Rafat, Alexis Garduno, Rachael Cusack, Jesus Francisco Bermejo-Martin, Ignacio Martin-Loeches, David Kelvin

**Affiliations:** ^1^ Laboratory of Emerging Infectious Diseases, Department of Immunology and Microbiology, Dalhousie University, Halifax, NS, Canada; ^2^ Department of Pediatrics, Izaak Walton Killan (IWK) Health Center, CCfV, Halifax, NS, Canada; ^3^ Department of Clinical Medicine, Trinity College, University of Dublin, Dublin, Ireland; ^4^ Instituto de Investigación Biomédica de Salamanca (IBSAL), Gerencia Regional de Salud de Castilla y León, Paseo de San Vicente, Salamanca, Spain; ^5^ Universidad de Salamanca, C. Alfonso X el Sabio, s/n, Salamanca, Spain; ^6^ Centro de Investigación Biomédica en Red en Enfermedades Respiratorias (CIBERES), CB22/06/00035, Instituto de Salud Carlos III, Avenida de Monforte de Lemos, Madrid, Spain

**Keywords:** biomarkers, data mining, pattern recocgnition, artificial intelligence, COVID - 19, sepsis, septic shock

## Abstract

**Introduction:**

Millions of deaths worldwide are a result of sepsis (viral and bacterial) and septic shock syndromes which originate from microbial infections and cause a dysregulated host immune response. These diseases share both clinical and immunological patterns that involve a plethora of biomarkers that can be quantified and used to explain the severity level of the disease. Therefore, we hypothesize that the severity of sepsis and septic shock in patients is a function of the concentration of biomarkers of patients.

**Methods:**

In our work, we quantified data from 30 biomarkers with direct immune function. We used distinct Feature Selection algorithms to isolate biomarkers to be fed into machine learning algorithms, whose mapping of the decision process would allow us to propose an early diagnostic tool.

**Results:**

We isolated two biomarkers, i.e., Programmed Death Ligand-1 and Myeloperoxidase, that were flagged by the interpretation of an Artificial Neural Network. The upregulation of both biomarkers was indicated as contributing to increase the severity level in sepsis (viral and bacterial induced) and septic shock patients.

**Discussion:**

In conclusion, we built a function considering biomarker concentrations to explain severity among sepsis, sepsis COVID, and septic shock patients. The rules of this function include biomarkers with known medical, biological, and immunological activity, favoring the development of an early diagnosis system based in knowledge extracted from artificial intelligence.

## 1 Introduction

Sepsis and septic shock are life-threatening syndromes that are associated with dysregulation in the host immune responses to infection (1). They can lead to organ failure and consequently death (2). As an example, in 2017, more than 11 million deaths associated with sepsis were reported worldwide (3), which represented a mortality rate of approximately 22%. Moreover, a considerable share of viral sepsis patients (i.e., sepsis COVID) meet the definition for sepsis-3 (bacteria-induced sepsis) (4). A more severe manifestation of sepsis is septic shock, in which patients meet all sepsis-3 criteria and require the use of vasopressor (1).

The fact that sepsis (of viral and bacterial sources) and its severe subset (i.e., septic shock) meet the criteria for sepsis-3 definition allows the employment of biomarkers as an early diagnostic tool (5). We selected a consortium composed of the following 30 biomarkers that are responsible for reflecting signals of specific moments during an immune response towards a pathogen that causes sepsis and/or septic shock: Angiopoietin 2 (ANG2), C-C Chemokine Ligand 2 (CCL2), C-X-C Motif Chemokine Ligand 10 (CXCL10), D-dimer, E-selectin (E-SEL), ferritin, Granulocyte Colony-Stimulating Factor (G-CSF), Granulocyte Macrophage Colony-Stimulating Factor (GM-CSF), Granzyme B (GRANB), Intercellular Adhesion Molecule 1 (ICAM-1), Interferon ɣ (IFNɣ), Interleukin 1 β (IL1β), Interleukin 1 receptor antagonist (IL1ra), Interleukin 2 (IL2), Interleukin 4 (IL4), Interleukin 6 (IL6), Interleukin 7 (IL7), Interleukin 10 (IL10), Interleukin 12 (IL12), Interleukin 15 (IL15), Interleukin 17 (IL17a), Lipocalin-2 (LIPO), Myeloperoxidase (MPO), Programmed Death-Ligand 1 (PDL1), Soluble glycoprotein 130 (sGP130), Soluble interleukin 6 receptor (sIL6R), Surfactant Protein (SPD), Tumor Necrosis Factor-alpha (TNF-ɑ), Vascular Cell Adhesion Molecule 1 (VCAM), and Vascular Endothelial Growth Factor C (VEGFC). Further details on the impact of the dysregulation of these biomarkers are shown in **Supplementary Material S1**.

The inflammatory pathway of the diseases explored here leaves traces behind that might be employed in profiling the severity of the diseases themselves (6, 7). Many of these traces are depicted through the analysis of biomarkers (i.e., cytokines and chemokines) associated with the host immune system. For example, VCAM-1, ICAM-1, and VEGFC are recruited when there is damage to vascular tissue (8). Moreover, CCL2 orchestrates the recruitment of immune cells to sites of inflammation (9). In addition, PDL1 functions as a suppressor of the adaptive immune system as it binds to the Programmed Cell Death Protein 1 (PD1) (10). Finally, MPO is mainly expressed in neutrophil granulocytes, granting antipathogenic activity to the immune cells expressing them (11). Each biomarker has an individual and grouped function in inflammatory pathways (12); therefore, a systematic analysis using data mining to determine the key items involved in sepsis/septic shock syndrome is appreciated (13, 14).

It has been stated (15) that a dataset comprised of too many dimensions (i.e., biomarkers) might slow, mask, and reduce the efficiency of machine learning approaches. Therefore, the selection of the most important features of a dataset is an essential step within data pre-processing frameworks. Moreover, Explainable Artificial Intelligence (XAI) has arisen as a manner to promote comprehension for the decision pattern employed by machine learning approaches, especially with the solid ethical standards required by the medical sciences. Therefore, decision-making processes benefit of a mathematically evidenced procedure (16).

In this paper, we hypothesize that the severity of sepsis (bacterial and viral) and septic shock patients is a function of the concentration of biomarkers. For that, we aim to use feature selection algorithms that will isolate biomarkers as candidates for distinguishing the severity of multi-organ failure in sepsis, sepsis COVID, and septic shock patients. With subsets of the selected biomarkers, we aim to evaluate these subsets through interpretable Artificial Neural Networks, so the biomarker concentration that defines the severity of patients with sepsis, sepsis COVID, and septic shock can be used as an early diagnostic tool.

## 2 Materials and methods

### 2.1 Study design

All the samples used in this study were obtained from a critically ill cohort of Intensive Care Unit (ICU) sepsis, sepsis COVID, and septic shock patients at St James’s Hospital in Dublin, Ireland. Institutional Research Board approval was granted by the SJH/TUH Joint Research Ethics Committee and The Health Research Consent Declaration Committee (HRCDC) under the register number REC: 2020-05 List 17 and project ID 0428. Biological samples, clinical findings, and laboratory data were collected at days 0, 3, and 14 after presentation of severe infection to monitor the progression and sepsis-induced immune-paralysis state at different stages of the disease. Sample collection took place from September 2020 to March 2021. Sequential Organ Failure Assessment (SOFA) score was obtained on admission to the ICU and at the matching collection timepoints for samples. The clinical variables for white blood cell (WBC) count (worst record of day 0), neutrophils (day 0), positive culture, and up to five comorbidities were attributed to each patient.

### 2.2 Biomarker immunoassays

The concentration of biomarkers with potential altered functions in sepsis, sepsis COVID, and septic shock (10) patients was quantified in the Laboratory of Emerging Infectious Diseases at Dalhousie University in Halifax, Nova Scotia, Canada. The following biomarkers were quantified through the Ella SimplePlex Immunoassay™ (San Jose, California): ICAM-1, LIPO, MPO, VCAM-1, D-Dimer, E-SEL, Ferritin, SPD, PDL1, G-CSF, IL-1b, VEGFC, ANG2, CXCL10, GM-CSF), Interleukin 10 (IL-10, IL-17A, IL-1ra, IL-6, IL-7, CCL2, GRANB, IFNg, IL-12, IL-15, IL-2, IL-4, and TNF-α. The biomarkers were selected due to their potential as characterizing the patients’ inflammation (12).

The plasma concentration of both sIL6R and sGP130 was evaluated and quantified with Enzyme-Linked Immunosorbent Assay (ELISA) kits (BMS214TEN for sIL-6R and EHIL6STX10 for sGP130; ThermoFisher Scientific). These biomarkers were selected due to their crucial role in the IL-6 inflammatory pathway (17). Finally, each sample was assayed and quantified following the kit manufacturer’s instructions. All samples were obtained from patients already admitted to the intensive care unit (ICU). We included the concentrations for all biomarkers at day zero of ICU in **Supplementary Material S2**. We provide the dataset in **Supplementary Material S2**.

### 2.3 Binarization of SOFA score into different degrees of multi-organ failure

Each patient in our cohort was assigned a Sequential Organ Failure Assessment (SOFA) score. For classification purposes, we binarized the SOFA score into two groups, i.e., High Degree Multi-Organ Failure (HDMOF) and Low Degree Multi-Organ Failure (LDMOF). We employed a cut-off value of 8, as reported by (18) to binarize the groups; thus the HDMOF group is characterized by a SOFA score equal to or higher than 8 while the LDMOF group has a SOFA score less than 8. In their paper, upon binarizing patients with a SOFA score cut of (>=8, and<8) Martin-Loeches et al. (2017) (18) found different mortality rates and antibodies levels that well explained the severity of sepsis patients.

### 2.4 Statistical analyses

All statistical procedures were performed using R. We used the Shapiro-Wilk test to find data distribution. To compare averages between groups, the Kruskal-Wallis test, t test, and ANOVA test were used through the Rstatix package (version 0.7.0). The Kaplan-Meier method under the survival R package (version 3.3-1) was used for calculating survival probabilities for each group (i.e., sepsis, sepsis COVID, and septic shock). The level of significance was set to 0.05.

### 2.5 Feature selection

To systematically choose biomarkers that are associated with high and low MOF, a Feature Selection (FS) step was applied, and the outcomes were benchmarked. The FS algorithms chosen for this study are representatives of three distinct classes of these methods. For a wrapper algorithm, we selected Boruta and applied the parameters specified in (19); the filter algorithm we chose is Information Gain (IG); and a combination of both FS classes, which results in an embedded algorithm has as its representative the Lasso Regression (LR) under the parameters specified in (20). Both Boruta and LR were implemented in R through the packages Boruta (version 7.0.0) and Glmnet (version 4.1-4), respectively. IG was implemented in Python using the class mutual_info_classif found in the sklearn.feature_selection library (version 1.1.0). Both the R and Python scripts that performed the feature selection process are available at https://github.com/gustavsganzerla/covid-biomarker/blob/main/XAI/feature_selection.R and https://github.com/gustavsganzerla/covid-biomarker/blob/main/XAI/information_gain.py, respectively.

### 2.6 Classification of severity with artificial intelligence

We selected the algorithms Support Vector Machines (SVM), Random Forest (RF), Classification and Regression Trees (CART), K-Nearest Neighbors (KNN), and deep learning Artificial Neural Networks (ANN) to classify patients’ severity with immune biomarkers as input. For that, we performed a 10-fold cross-validation process on the input data. The test dataset encompassed 20% of the whole data. The first five classification algorithms were implemented in R through the Caret package (version 6.0-9) and their code is available at https://github.com/gustavsganzerla/covid-biomarker/blob/main/XAI/classific.R.

The ANN approach was developed in Python using the Tensorflow library (version 2.8.0). A sigmoid function was used in the output layer to return a probability of a given patient having MOF or not. The outcome was binarized through a confusion matrix built under the default 0.5 decision threshold applied in the outcome of the output neuron. The classes obtained were categorized as follows: True Positives (TPs), True Negatives (TNs), False Positive (FPs), and False Negatives (FNs). The ANN performance was evaluated in terms of accuracy, AUC, FPs, FNs, TPs, TNs, all of which can be found in the module *tf.keras.metrics*. The architecture of the ANN has input layers that vary according to the set of biomarkers entered. Next, the model has two fully connected layers with 10 hidden neurons each. We also increased the epochs of the ANN until the error (binary cross entropy) kept dropping. The scripts containing the ANN simulation are available at https://github.com/gustavsganzerla/covid-biomarker/blob/main/XAI/SHAP-ANN.

### 2.7 Explanation of the classification model

We used Shapley Additive Explanations (SHAP) (21) to provide interpretability to the successful classification models. SHAP will assign a score (either positive or negative) for each input variable in assigning a label to an observation. If the SHAP score of a given feature is positive, it is positively correlated with the assigning of a label; otherwise, it is negatively correlated with the target label.

The SHAP approach is defined as a solid theoretical foundation that may be used to explain any predictive model locally and globally. From this, we employed the *kernel.explainer* method in the SHAP module. As our classifier is not a tree-based algorithm, the Kernel SHAP is applied. Kernel SHAP will measure the contribution of each input feature to the outcome of the model, it consists of five steps:

- Sample all possible coalitions (i.e., combination of input features) in the dataset: $Z_{K}^{'}\in {\left\{0,1\right\}}^{M},\;K\;\in \left\{1,\ldots ,\;K\right\}$ . (0 = feature absent and 1 = feature present in the coalition).
- The prediction of each *Z’_K_* is obtained with the application of *Z’_K_* to the predictive model.
- The weight of each *Z’_K_* is computed by the SHAP kernel.
- The model is fitted.
- Return the SHAP coefficients *ϕ_i_.*

**Figure 1:**
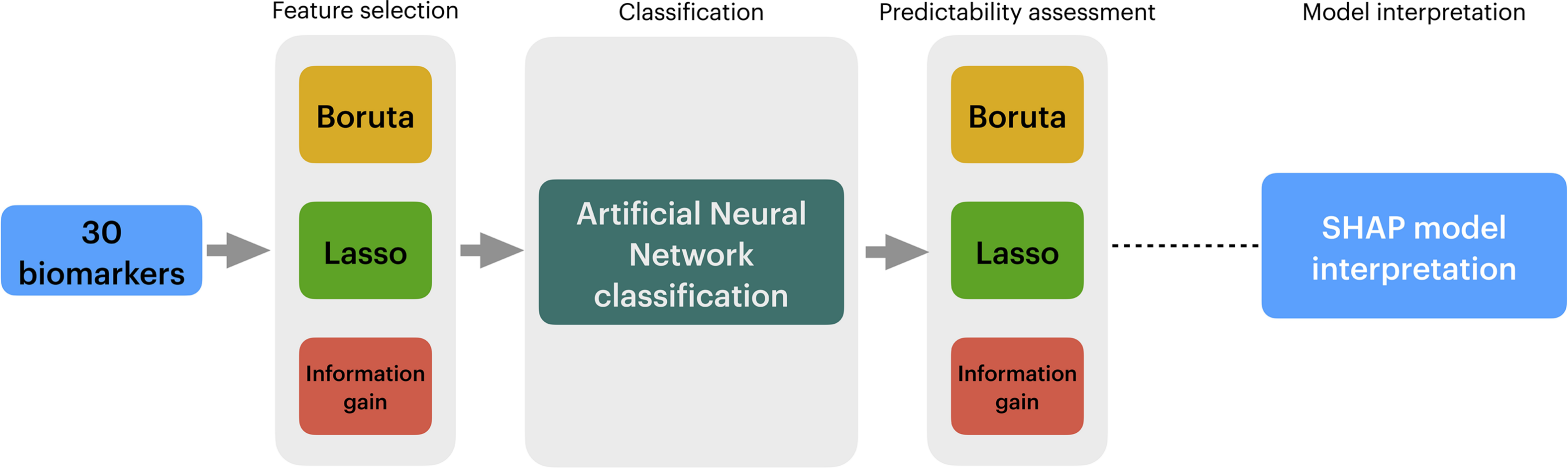
Overview of the data analysis procedure employed in this study. In **Figure 1**, we show the three stages of the data analytical process employed in this study. First, 30 biomarkers from sepsis, sepsis COVID, and septic shock patients were obtained. Secondly, to reduce the number of variables, three algorithms are applied in the Feature Selection stage, i.e., Boruta, Lasso Regression, and Information Gain. The full outcome of the three algorithms is classified into an Artificial Neural Network (ANN). A second filter is applied to promote more reduction to the data using Exhaustive Search, whose outcomes are yet fed into ANNs in to compare their performance with the full outcomes of Boruta, Lasso Regression, and Information Gain. After running multiple ANNs, the prediction model is evaluated with SHAP.

In conclusion, A flowchart describing the data analytical process employed in this study is available in **Figure 1**.

## 3 Results

### 3.1 Clinical characteristics and survival analysis

We provide **Table 1** to clinically depict the population that composes the cohort (n=112). We identify that most patients in the cohort are male (60%) with an average age of 64.7 years old. The average ICU stay was 38.3 days. Finally, 63.7% of all patients survived while the lowest reported mortality was in sepsis (30%) patients followed by sepsis COVID (32.4%) patients and septic shock patients (38%). We also report that the patients with septic shock have a higher count of neutrophils. Differences in the neutrophil counts were found to be associated with the severity of the disease; i.e., in the severe form of sepsis, the leukocytes count increased 1.62-fold and the increase in sepsis patients was 1.41-fold, while the leukocytes count remained more stable in septic shock (1.005-fold) patients. Next, we report the differential WBC count in sepsis, sepsis COVID, and septic shock (1.44, 1.15, and 1.08, respectively). Patients were assessed according to positive microbiological culture. We report that the COVID patients showed a smaller proportion of patients with viral and bacterial co-infection, i.e., superinfection (9 patients, 7 in LDMOF and 2 in HDMOF). The positive culture results for patients without COVID was found stable across both groups in sepsis and septic shock. Finally, we found the most common comorbidities to be hypertension, affecting 45% of the entire population of the cohort, followed by obesity (18%), and chronic obstructive pulmonary disease (15%). At the time of the study (i.e., September 2020 to March 2021), the circulating COVID-19 variant in the British Isles was B.1.1.7.

**Table 1:** Clinical characteristics of the cohort.

	Sepsis		Sepsis COVID		Septic shock		All patients
	LDMOF	HDMOF	LDMOF	HDMOF	LDMOF	HDMOF	
n (%)	21	9	28	22	18	14	112
Age (mean ± standard deviation)	58.2 ± 10	65.2 ± 15	62.4 ± 10	68.3 ± 10	63 ± 15	69.1 ± 16	64.7 ± 12.8
Female (n)	7	4	12	10	8	3	44
Male (n)	11	8	11	17	8	13	68
ICU stay (days, mean)	27	25	25	53	16	19	38.3
Survived [n (%)]	18 (85%)	3 (33.3%)	21 (75%)	10 (45.4%)	13 (72.2%)	6 (42.8%)	71 (63.3%)
SOFA score (mean)	4.5	10.4	4.6	10.2	2.9	12	7.6
White blood cells	13.1	18.8	11.7	13.5	23	24.9	16.5
Neutrophil count (mean)	9.6	15.6	9	12.7	18.6	18.7	13.8
Lymphocyte count (mean)	1.2	0.9	0.8	0.8	2.1	2.7	1.3
Positive culture (%)^1^	66.6%	66.6%	27%	9%	66.6%	57%	49%
Coinfection (sars-cov-2 + bacteria) (%)	–	–	21%	36%	–	–	–
Comorbidities (n [%])							
Hypertension	9 (43%)	2 (22%)	8 (28.5%)	12 (54.5%)	7 (39%)	3 (21.5%)	39 (35%)
Cancer	2 (9.5%)	1 (11%)	1 (3.5%)	2 (9%)	3 (17%)	6 (43%)	15 (13.5%)
Asthma	1 (5%)	0 (0%)	6 (21.5%)	4 (18%)	1 (5.5%)	2 (14%)	14 (12.5%)
Diabetes mellitus	1 (5%)	0 (0%)	4 (14%)	6 (27%)	0 (0%)	4 (28.5%)	15 (13.5%)
Obesity	2 (9.5%)	2 (22%)	8 (28.5%)	3 (13.5%)	5 (27.5%)	0 (0%)	20 (18%)
Chronic obstructive pulmonary disease	5 (24%)	3 (33%)	5 (18%)	2 (9%)	0 (0%)	2 (14%)	17 (15%)

^1^Patients labeled with positive culture indicate bacteria present in their samples. The most common microorganisms are Staphylococcus epidermidis (n=11), Pseudomonas aeruginosa (n=7), Enterococcus faecalis (n=6), Escherichia coli (n=5), Klebsiella pneumoniae (n=2). The full description of the microbiology of each patient is included in **Supplementary Material S2**. "-" means that the information was not available.

**Figure 2:**
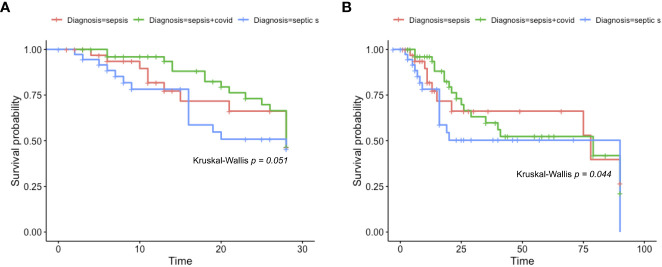
Kaplan-Meier survival rate. Kaplan-Meier survival probabilities were identified. In **(A)**, we show the Kaplan-Meier survival rate of sepsis, sepsis COVID, and septic shock patients after 28 days. Firstly, the data was identified as non-parametric (Shapiro-Wilk *p* = 1.377e-08) and the Kruskal-Wallis test was chosen to compare the averages (*p =* 0.051). In **(B)**, we show the Kaplan-Meier curve for sepsis, sepsis COVID, and septic shock after 90 days, the data also follows a non-parametric distribution (Shapiro-Wilk = 6.036e-11) and the same Kruskal-Wallis test was employed to compare the averages (*p* = 0.044).

We also employed a survival analysis by days 28 and 90 (**Figure 2**) to assess the mortality probability of sepsis, septic shock, and sepsis COVID patients. First, in **Figure 2A**, up to 28 days, the three groups of patients did not present significant differences in their survival probability (*p*=0.051); however, the low *p* value indicates a trend among the three groups’ survivability rate, placing septic shock as the highest mortality rate after 28 days. When a 90-days analysis was considered (**Figure 2B**), the survival rate among the patients showed statistical significance (*p* = 0.044), where the septic shock group presented the lowest survival probability and sepsis the highest.

Finally, to validate the binarization we performed, described in (18), we selected clinical parameters in our data that each, individually, represent the failure of a single organ. By following a logical expression that considers vasopressor as an exclusive (conjunction, AND) variable and platelets, bilirubin, creatinine, and P/F ratio as inclusive (disjunction, OR), our binarization resulted in an AUROC score of 0.91 followed by a Youden index of 0.71 (**Supplementary Material S3**).

### 3.2 Outcome of feature selection algorithms

**Figure 3:**
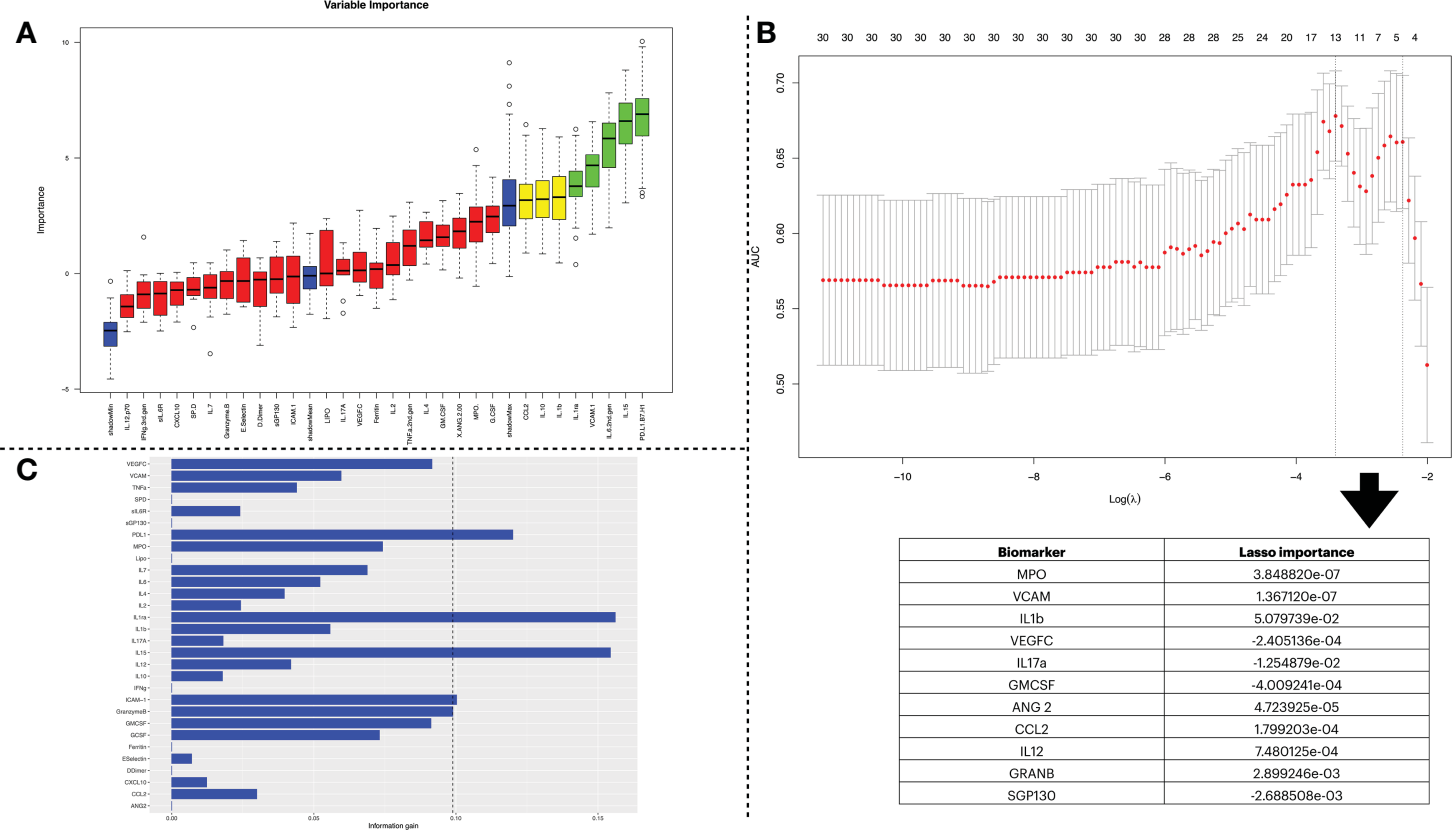
Biomarker selection using three feature selection algorithms. **(A)** indicates the feature selection process using the Boruta algorithm. The first set of obtained variables was submitted to a tentative fix method to deliver a more reliable subset. In the plot, the columns shown in green are the ones confirmed by the algorithm to be statistically significant and have higher importance in describing the data’s label. **(B)** shows the results obtained by the feature selection using Lasso Regression. The x-axis of the figure indicates the log of lambda. Since Lasso Regression might be used as a classification model, the y-axis shows the AUC when including the number of variables shown at the top of the plot. The table below shows the variables stored in the *lasso$lambda.min* object, which corresponds to the variables with the best lambda values. The more distant a value is from zero, the more relevant it is for the predictor once that Lasso Regression sets the lambda = 0 to unimportant variables. **(C)** conveys the information gain derived from dataset entropy reduction achieved by the Information Gain algorithm. The vertical dashed line represents a threshold that considered the five most impactful biomarkers.

To select a subset of biomarkers that explain the target variables (i.e., LDMOF and HDMOF), we performed a feature selection process. We chose three algorithms of distinct classes, namely Boruta, LR, and IG. Each application returned a different subset of biomarkers with varied lengths. We show in **Figure 3**, through panels A, B, and C, the outcomes of the Boruta, LR, and IG algorithms, respectively. The subsets of biomarkers obtained are as follows: *i*) PDL1, IL15, IL6, VCAM, IL1ra, IL1b, IL10, and CCL2 in Boruta; *ii*) MPO, VCAM, IL1b, VEGFC, IL17a, GMCSF, ANG2, CCL2, IL12, GRANB, and SGP130 in LR; and *iii*) PDL1, GRANB, IL15, ICAM, and IL1ra in IG.

### 3.3 Assessing the classification capacity of groups of biomarkers with different algorithms

We assessed the classification feasibility with five different machine learning algorithms, i.e., SVM, RF, CART, KNN, and ANN. We fed the classification models with the input variables identified by each one of our three FS algorithms (**Table 2**). From that, we identified that the ANNs presented the most satisfactory predictability as its accuracy score outperformed the other methods.

**Table 2:** Benchmarking different classification algorithms.

	Boruta (%)	Lasso Regression (%)	Information Gain (%)
CART	58	57.6	64.66
KNN	58.33	58	56.66
SVM	57.33	58	58.33
RF	63.66	59.33	64.66
ANN	96	96.2	78.3

**Figure 4:**
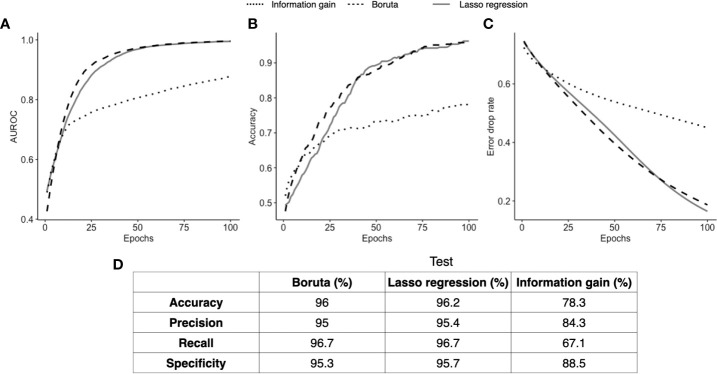
ANN classification performance. Three ANN models fed with different input biomarkers had their classification performance assessed over 100 learning epochs (Boruta in green, Lasso regression in orange, and information gain in blue). In **(A)**, we compare the area under the curve of the three models; in **(B)** we show the accuracy of each model; and in **(C)** we show the error drop rate (represented by binary cross entropy). Finally, in **(D)**, we show at the 100^th^ epoch the accuracy, precision, recall, and specificity of each model.

**Figure 5:**
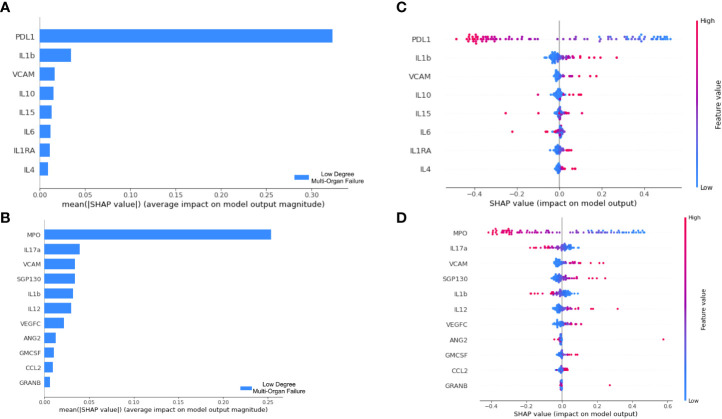
Decision pattern of ANNs classifying patients’ severity with different input biomarkers. The importance of each input biomarker determined by SHAP is displayed in **(A, B)** for the ANN derived of Boruta and Lasso Regression, respectively. In **(C, D)** we track the decision of two ANN models in classifying patients. For that, each biomarker is included in the y axis; each patient is represented by a dot in the plot, which has a high or low value corresponding to the biomarker concentration. Finally, the longitudinal location of the dots in the x axis indicates the impact in the SHAP value. Positive SHAP values are used to explain our target class, i.e., LDMOF while negative SHAP values represent the opposite class (i.e., HDMOF).

The results displayed in **Figure 4** indicate the detailed performance of the ANN models. To identify the best performing subset of biomarkers, we selected the model that presented the best error drop rate after 100 epochs, area under the curve (AUC), accuracy, precision, specificity, and recall. Next, the selected model was trained and tested with the subsets of biomarkers obtained in the FS stage. From that, the IG algorithm did not produce satisfactory results due to the imbalance of its prediction capacity, in which a low recall value was found (67.1%), indicating that it identified too many FNs in proportion of TPs, which is explained by the high error drop rate of the ANN model. Conversely, the ANNs trained/tested with the outcomes of LR and Boruta yielded satisfactory results, which is observed by the proximity of AUC, accuracy, and error drop rate (**Figures 4A–C**) as well as the balanced metrics provided for the models in **Figure 5D**. Therefore, ANNs successfully distinguished the severity of patients with the consortium of input biomarkers: *i*) PDL1, IL15, IL6, VCAM, IL1ra, IL10, IL1b, IL4, and *ii*) MPO, VCAM, IL1b, VEGFC, IL17a, GMCSF, ANG2, CCL2, IL12, GRANB, and SGP130.

### 3.4 Model interpretation

To interpret the decision pattern of each ANN model, a SHAP approach was applied to the training dataset. Since the ANN trained/tested with the biomarkers obtained by the Information Gain approach did not produce satisfactory classification metrics, we opted not to interpret this erroneous classification model. For both Boruta and Lasso regression, we analyzed the SHAP value for each input biomarker. We targeted the LDMOF class out of our train dataset and checked the contribution of each biomarker in predicting the class. First, in **Figures 5A, B**, we show that both PDL1 and MPO were the biomarkers that mostly contributed to predictions in their models.

Next, in **Figures 5C, D**, we demonstrate the concentration of each biomarker in assigning the LDMOF label for each patient. From that, we see that there is a clear division between patients with high/low concentrations of input biomarkers (blue and red dots, representing each patient of the train set) getting negative and positive SHAP values, which directly affects the label assignment. To provide individual explanations for each biomarker, we targeted the ones that are positively correlated with LDMOF (i.e., VCAM, IL1ra, and IL4) and the biomarkers that are negatively correlated with HDMOF (i.e., PDL1, MPO, IL17a, and VEGFC). The remaining biomarkers did not have a clear separation between our target variables. Additionally, our two ANN models did not produce the same results regarding IL1b since it had different behaviors in each model. Therefore, the XAI approach of our tool enabled us to locate biomarkers with pro- and anti-inflammatory activities.

### 3.5 Conserved programmed death Ligand-1 and myeloperoxidase signals across patients with distinct manifestations of organ failure syndromes

**Figure 6:**
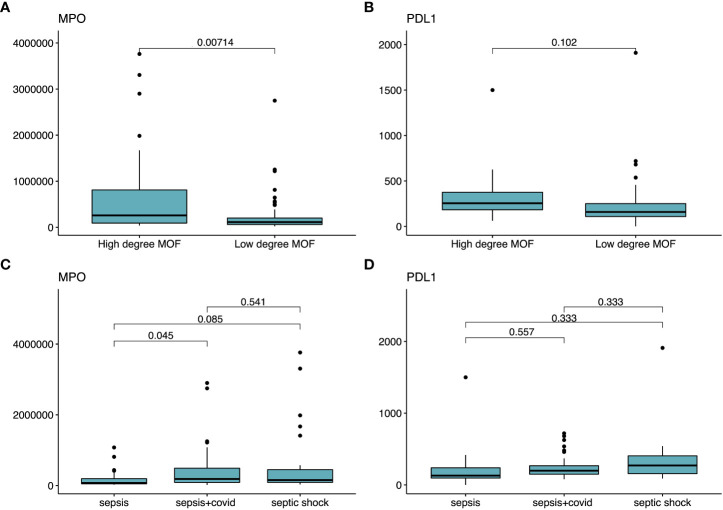
Statistical tests employed for PDL1 and MPO as characterizers of severity and disease model. Statistical significance tests comparing the averages of different groups of patients. In **(A, B)**, we show the mean comparison of MPO and PDL1 (respectively) in distinguishing each one of the target labels (i.e., HDMOF and LDMOF). In **(C, D)**, we employ the mean concentration MPO and PDL1 (respectively) in distinguishing sepsis, sepsis COVID, and septic shock.

We selected the two biomarkers flagged by the ANN (i.e., MPO and PDL1) to look for statistical differences between biomarker concentration and *i*) the three diseases of our cohort (sepsis, septic shock, and sepsis COVID-19) and *ii*) the two severity levels our binarization considered (LDMOF and HDMOF) (**Figure 6**). Statistical significance (*p* = 0.007) was found only in the distinction of the MPO concentration among HDMOF and LDMOF patients (**Figure 6A**), while the severity groups could not be statistically explained in terms of their PDL1 concentration (**Figure 6B**). We only found statistical difference (*p* = 0.045) in using MPO as a distinguisher of sepsis and sepsis COVID (**Figure 6C**), while the PDL1 concentration could not statistically differentiate sepsis, sepsis COVID, and septic shock (**Figure 6D**).

## 4 Discussion

In our study, we could explain the severity of sepsis, sepsis COVID, and septic shock patients as a function of an unbalanced concentration of biomarkers. The input parameters of our function are subsets of biomarkers that explain a dysregulated host immune response. Moreover, we could isolate both MPO and PDL1 as the key contributors to the function.

The output parameters of our function are high and low degree of multi-organ failure (i.e., severity) of subgroups of patients that all meet sepsis-3 criteria, clinically placing the patients into a wider group, converging with past evidence (4). Our findings allowed us to immunologically place the patients from different disease models together as we failed to find major statistical significances in the concentration of PDL1 and MPO (**Figures 6C, D**) that distinguish sepsis, sepsis COVID, and septic shock all together. The only differences we could observe were in the MPO concentration between sepsis COVID and sepsis patients, and we argue that this is a result of the generally higher neutrophil count in bacterial induced sepsis (22). Finally, the SOFA score-based binarization we achieved is on par with results previously reported (18).

Next we showed that the WBC count increased with severity. Neutrophils, the most abundant WBC, were higher in the severe manifestations of the diseases we assessed. Moreover, the WBC and neutrophil count was lower in COVID sepsis, matching previous references of neutrophils being one of the most responsive cells toward bacterial infection (22). We argue that the lower occurrence of bacterial infection found in COVID (i.e., superinfection) patients contributed to their lower count of WBC and neutrophils. Nonetheless, neutrophils remained an important immune cell to express cytokines, which might explain their response toward infection. Furthermore, no records of comorbidities influencing severity were found, except from cancer; we found the proportion of patients with cancer was higher in the severe form of the three disease models we assayed. The two key biomarkers we found (i.e., MPO and PDL1) are parts of important pathways in cancer immunotherapies (23) providing an opportunity for future studies of data science approaches for biomarkers involved in cancer.

Statistically, no significant differences were found that distinguished our patients based on their severity (**Figures 6A, B**). It was previously reported that when statistics do not reach a satisfactory classification performance, Machine Learning (ML) might be a valid approach (24). We tried different ML approaches to classify our data. From five algorithms tested, only ANNs yielded a satisfactory classification. In fact, the robustness of this method was previously reported (24) as a solid way to find patterns in tabular data, among others. Additionally, the appropriate selection of the input variables is a key process in obtaining satisfactory results (25). In many cases, classification and regression models derived from lower-dimensional datasets benefit the downstream decision-making process (26, 27). We further address this discussion by linking the appropriate selection of biomarkers with easily interpretable results in an information curation step. In this study, we were able to reduce a total of 30 biomarkers using three FS algorithms into subsets that conveyed satisfactory classification results in two instances. The three algorithms we selected belong to diverse classes of FS methods. In fact, they all have successfully been applied in reducing the complexity of ML inputs (20, 28–32) without creating synthetic datasets. The results we obtained highlight the reduced complexity in employing data-preprocessing (DP) techniques. In fact, DP accounts for most of the workload involved in ML applications (33). We link the lack of success of the information gain algorithm in producing satisfactory predictability to the fact that this algorithm will only look for the association between input variables with a label (i.e., biomarkers and LDMOF/HDMOF). The other two FS algorithms will fit a model to determine the importance of each individual variable in predicting a label, granting them mathematical robustness. Therefore, we argue that a systematic evaluation of DP techniques such as the one here proposed is highly beneficial for developing *in-silico* models.

After selecting optimal input biomarkers, we applied them in a classification system that uses this immunological information to predict the severity trajectory of critically ill patients. For that, our model, when fed with distinct subsets of biomarkers, could predict patients’ severity. In fact, biomarkers have themselves been proposed as good predictors of a plethora of medical conditions (34–36); on the immunological side, they have been associated with pro- and anti-inflammatory responses (37) and we gathered evidence in support of our model succeeding to capture this. There are hundreds of biomarkers containing valuable information about the organic systems of the body and their functions. For their capacity to be interpreted, and consequently aid decision making and drug development, we argue that biomarkers related to a specific condition be systematically selected as we have proposed in this study.

We interpret our ANN model by linking the concentration of biomarkers in explaining LDMOF. First, Multiple Organ Dysfunction and even death have been reported to be associated with increased levels of VCAM-1 in adults and neonates diagnosed with sepsis (38–40). Similarly, our model shows a negative correlation between increased VCAM levels and LDMOF development. Next, IL1ra was used in a recombinant treatment and was successful in reducing levels of mortality (41). Our last biomarker positively associated with LDMOF is IL4, which was reported to act together with IL6 to induce Th2 cells and macrophage differentiation (42). Lower concentrations of this biomarker were associated with lower mortality of severe sepsis patients (43).

We spotted four biomarkers that are negatively correlated with LDMOF, granting them a negative effect on a patient’s favorable outcome. First, low concentrations of IL17a have been reported as a good predictor for mortality in sepsis caused by distinct pathogens (44, 45). Next, septic shock and sepsis can lead to hypoxia due to tissue hypoperfusion (1). A transcription factor named hypoxia-inducible factor 1-alpha (HIF-1α) accumulates in cells under hypoxic conditions and can upregulate the expression of VEGF and PD-L1 (46, 47). The activated HIF pathway can also trigger the activation of innate immune cells, including macrophages, dendritic cells, neutrophils, and natural killer cells (48). Furthermore, increased levels of PD-L1 will suppress the adaptive immune response by inhibiting the proliferation and activity of the CD4+ effector T cells and enhancing the differentiation of Tregs (49, 50). This persistent inflammatory condition caused by innate immune activation along with suppressed adaptive immune response contributes to hypoxia-induced organ failure. It has also been reported that PD-L1 knock-out animals show a better survival rate after a septic challenge compared to wild-type animals (51). VEGF is also known to induce VCAM-1 expression, and its upregulation is correlated with damaged vascular endothelium and organ dysfunction (40, 52). Thus high levels of PD-L1 and VEGF can be considered key markers of multi-organ failure. Finally, myeloperoxidase (MPO), an enzyme produced by neutrophils, is found to be increased in severe patients suffering from septic shock, despite having no significant difference in neutrophil count (53). In another study, MPO levels during the early stages of sepsis were found to be negatively correlated with patient survival (54).

The mapping of the XAI model provided meaningful information on the course of dysregulated immune responses and also converged with clinical interpretations regarding the neutrophil count of the disease models (i.e., neutrophils tend to increase with severity). A compelling example is the pro-inflammatory function our model attributed to MPO. This enzyme is primarily produced by the granulocytes of neutrophils. The overexpression of MPO generates harmful chemicals that have a detrimental effect on organ inflammation (11). Our model also identified PDL1 as a pro-inflammatory protein. The blockade of the binding between PDL1 and PD1 might inhibit lymphocytes from apoptosis. The upregulation of PDL1 by neutrophils is increased in sepsis as the higher migration of these cells might allow them to be trapped in the lung vasculature (55, 56). Therefore, with more neutrophils expressing PDL1, immunosuppressant effects start to occur with the death of neutrophils. The highest counts of neutrophils were found in the severe manifestations of the three disease models we investigated. Therefore, we can track the course of the multi-organ failure syndrome of our patients with increased neutrophils leading the overexpression of MPO and PDL1.

*In-silico* models are an efficient paradigm of experimentation. Compelling examples are found in big data-based applications that have been assisting in several areas in the medical sciences, such as predicting heart attacks (57), telediagnosis (58), and preventing disease outbreaks (59), among others. A guideline proposed by the Center for Drug Evaluation and Research (CDER) (60) shows that the development of therapeutics might initiate with screening characteristics that indicate biological processes. Here we propound to employ data science as an initial step for screening biomarkers, enabling the gathering of solid mathematical evidence for linking concentrations of biomarkers with patients’ severity.

## 5 Conclusions

In the present study, we built a function whose input is the concentration of biomarkers and the output is the level of severity of a patient. For our goal to be achieved, a systematic data mining procedure enabled us to identify the upregulation of PDL1 and MPO as good predictors of severity in sepsis (viral and bacterial induced) and septic shock patients. After interpreting the results both clinically and immunologically, we found that there is solid medical and biological evidence for why the upregulation of PDL1 and MPO is a major driver of severity. To this extent, we posit that data mining routines such as the one we proposed be used to identify the biomarkers that can function as part of an early diagnosis system.

## Data availability statement

The datasets presented in this study can be found in online repositories. The names of the repository/repositories and accession number(s) can be found in the article/**Supplementary Material**.

## Ethics statement

The studies involving human participants were reviewed and approved by Health Research Consent Declaration Committee (HRCDC) under the register REC: 2020-05 List 17 and project ID 0428. The patients/participants provided their written informed consent to participate in this study.

## Author contributions

GM, DK, IM-L, AG and AO designed the study. GM worked on the machine learning approach. GM prepared the figures. AO linked the machine’s decision pattern with immunological activity. AO, AA-R, and DK performed the immunoassays. AG, RC, and IM-L managed the clinical aspect of the study. GM, AO, AA-R prepared the draft version. All authors contributed to the article and approved the submitted version.

## References

[1] Hotchkiss RS Moldawer LL Opal SM Reinhart K Turnbull IR Vincent JL. Sepsis and septic shock. Nat Rev Dis Primers (2016) 2:16045. doi: 10.1038/nrdp.2016.45 28117397 PMC5538252

[2] Schoenberg MH Weiss M Radermacher P. Outcome of patients with sepsis and septic shock after ICU treatment. Langenbeck’s Arch Surg (1998) 383(1):44–8. doi: 10.1007/s004230050090 9627170

[3] Rudd KE Johnson SC Agesa KM Shackelford KA Tsoi D Kievlan DR. Global, regional, and national sepsis incidence and mortality, 1990–2017: analysis for the global burden of disease study. Lancet (2020) 395(10219):200–11. doi: 10.1016/S0140-6736(19)32989-7 PMC6970225 31954465

[4] Karakike E Giamarellos-Bourboulis EJ Kyprianou M Fleischmann-Struzek C Pletz MW Netea MG. Coronavirus disease 2019 as cause of viral sepsis: A systematic review and meta-analysis. Crit Care Med (2021) 49(12):2042–57. doi: 10.1097/CCM.0000000000005195 PMC8594513 34259663

[5] Von Groote T Meersch-Dini M. Biomarkers for the prediction and judgement of sepsis and sepsis complications: A step towards precision medicine? J Clin Med (2022) 11(19):5782. doi: 10.3390/jcm11195782 36233650 PMC9571838

[6] Rivers EP Kruse JA Jacobsen G Shah K Loomba M Otero R. The influence of early hemodynamic optimization on biomarker patterns of severe sepsis and septic shock. Crit Care Med (2007) 35(9):2016–24. doi: 10.1097/01.CCM.0000281637.08984.6E 17855815

[7] Lee EE Song KH Hwang W Ham SY Jeong H Kim JH. Pattern of inflammatory immune response determines the clinical course and outcome of COVID-19: unbiased clustering analysis. Sci Rep (2021) 11(1):8080. doi: 10.1038/s41598-021-87668-z 33850271 PMC8044143

[8] Zhao J Chen L Shu B Tang J Zhang L Xie J. Granulocyte/macrophage colony-stimulating factor influences angiogenesis by regulating the coordinated expression of VEGF and the Ang/Tie system. PloS One (2014) 9(3):e92691. doi: 10.1371/journal.pone.0092691 24658178 PMC3962430

[9] Kong DH Kim YK Kim MR Jang JH Lee S. Emerging roles of vascular cell adhesion molecule-1 (VCAM-1) in immunological disorders and cancer. Int J Mol Sci (2018) 19(4):1057. doi: 10.3390/ijms19041057 29614819 PMC5979609

[10] Han Y Liu D Li L. PD-1/PD-L1 pathway: current researches in cancer. Am J Cancer Res (2020) 10(3):727–42. PMC7136921 32266087

[11] Aratani Y. Myeloperoxidase: Its role for host defense, inflammation, and neutrophil function. Arch Biochem Biophys (2018) 640:47–52. doi: 10.1016/j.abb.2018.01.004 29336940

[12] Bermejo-Martin JF González-Rivera M Almansa R Micheloud D Tedim AP Domínguez-Gil M. Viral RNA load in plasma is associated with critical illness and a dysregulated host response in COVID-19. Crit Care (2020) 24(1):691. doi: 10.1186/s13054-020-03398-0 33317616 PMC7734467

[13] Reinhart K Meisner M Brunkhorst FM. Markers for sepsis diagnosis: What is useful? Crit Care Clinics (2006) 22(3):503–19. doi: 10.1016/j.ccc.2006.03.003 16893736

[14] Kim MH Choi JH. An update on sepsis biomarkers. Infect Chemother (2020) 52(1):1–18. doi: 10.3947/ic.2020.52.1.1 PMC7113456 32239808

[15] Berisha V Krantsevich C Hahn PR Hahn S Dasarathy G Turaga P. Digital medicine and the curse of dimensionality. NPJ Digital Med (2021) 4(1):153. doi: 10.1038/s41746-021-00521-5 PMC8553745 34711924

[16] Gunning D Stefik M Choi J Miller T Stumpf S Yang GZ. XAI-explainable artificial intelligence. Sci Robotics (2019) 4(37):369–88. doi: 10.1126/scirobotics.aay7120 33137719

[17] Baran P Hansen S Waetzig GH Akbarzadeh M Lamertz L Huber HJ. The balance of interleukin (IL)-6, IL-6soluble IL-6 receptor (sIL-6R), and IL-6sIL-6Rsgp130 complexes allows simultaneous classic and trans-signaling. J Biol Chem (2018) 293(18):6762–75. doi: 10.1074/jbc.RA117.001163 PMC5936821 29559558

[18] Martin-Loeches I Muriel-Bombín A Ferrer R Artigas A Sole-Violan J Lorente L. The protective association of endogenous immunoglobulins against sepsis mortality is restricted to patients with moderate organ failure. Ann Intensive Care (2017) 7(1):44. doi: 10.1186/s13613-017-0268-3 28429310 PMC5399013

[19] Kursa MB Rudnicki WR. Feature selection with the boruta package. J Stat Software (2010) 36(11):1–13. doi: 10.18637/jss.v036.i11

[20] Kumarage PM Yogarajah B Ratnarajah N. (2019). Efficient feature selection for prediction of diabetic using LASSO, in: 19th International Conference on Advances in ICT for Emerging Regions, ICTer 2019 - Proceedings, IEEE, Colombo, Sri Lanka. doi: 10.1109/ICTer48817.2019.9023720

[21] Lundberg SM Lee SI. A unified approach to interpreting model predictions. Adv Neural Inf Process Syst NIPS'17: Proceedings of the 31st International Conference on Neural Information Processing Systems (2017) 4768–77. doi: 10.5555/3295222.3295230

[22] Camp J. v. Jonsson CB. A role for neutrophils in viral respiratory disease. Front Immunol (2017) 8:550. doi: 10.3389/fimmu.2017.00550 28553293 PMC5427094

[23] Zeindler J Angehrn F Droeser R Däster S Piscuoglio S Ng CKY. Infiltration by myeloperoxidase-positive neutrophils is an independent prognostic factor in breast cancer. Breast Cancer Res Treat (2019) 177(3):581–9. doi: 10.1007/s10549-019-05336-3 31267330

[24] Martinez GS Pérez-Rueda E Sarkar S Kumar A de Avila Silva S. Machine learning and statistics shape a novel path in archaeal promoter annotation. BMC Bioinf (2022) 23:171. doi: 10.1186/s12859-022-04714-x PMC9087966 35538405

[25] Martinez GS Garduno A Abdullah-Mahmud R Ostadgavahi AT Avery A de Avila e Silva S. An artificial neural network classification method employing longitudinally immune biomarkers to predict the clinical outcome of critically ill COVID-19 patients. PeerJ (2022) 10:e14477. doi: 10.7717/peerj.14487 36530391 PMC9753745

[26] Cai J Luo J Wang S Yang S. Feature selection in machine learning: A new perspective. Neurocomputing (2018) 300:70–9. doi: 10.1016/j.neucom.2017.11.077

[27] Liu H Motoda H. Feature selection for knowledge discovery and data mining. In: Feature selection for knowledge discovery and data mining. Springer New York, NY (1998). doi: 10.1007/978-1-4615-5689-3

[28] Ueno D Kawabe H Yamasaki S Demura T Kato K. Feature selection for RNA cleavage efficiency at specific sites using the LASSO regression model in arabidopsis thaliana. BMC Bioinf (2021) 22(1):380. doi: 10.1186/s12859-021-04291-5 PMC8299621 34294042

[29] Muthukrishnan R Rohini R. LASSO: A feature selection technique in predictive modeling for machine learning, in: 2016 IEEE International Conference on Advances in Computer Applications, ICACA 2016, IEEE, Coimbatore, India. (2017). doi: 10.1109/ICACA.2016.7887916

[30] Kursa MB Jankowski A Rudnicki WR. Boruta - a system for feature selection. Fundamenta Informat (2010) 101(4):271–85. doi: 10.3233/FI-2010-288

[31] Zhang S Zhang C Yang Q. Data preparation for data mining. Appl Artif Intell (2003) 17(5–6):375–81. doi: 10.1080/713827180

[32] Gao Z Xu Y Meng F Qi F Lin Z. Improved information gain-based feature selection for text categorization, in: 2014 4th International Conference on Wireless Communications, Vehicular Technology, Information Theory and Aerospace and Electronic Systems, VITAE 2014 - Co-Located with Global Wireless Summit, IEEE, Aalborg, Denmark. (2014). doi: 10.1109/VITAE.2014.6934421

[33] Huang J Li YF Xie M. An empirical analysis of data preprocessing for machine learning-based software cost estimation. Inf Software Technol (2015) 67:108–27. doi: 10.1016/j.infsof.2015.07.004

[34] Peter TJ Somasundaram K. Study and development of novel feature selection framework for heart disease prediction. Int J Sci Res Publications (2012) 2(10).

[35] Ridker PM. Role of inflammatory biomarkers in prediction of coronary heart disease. Lancet (2001) 358(9286). doi: 10.1016/S0140-6736(01)06112-8 11583743

[36] Velotti F Barchetta I Cimini FA Cavallo MG. Granzyme b in inflammatory diseases: Apoptosis, inflammation, extracellular matrix remodeling, epithelial-to-Mesenchymal transition and fibrosis. Front Immunol (2020) 11:587581. doi: 10.3389/fimmu.2020.587581 33262766 PMC7686573

[37] Leuchter AF Cook IA Hamilton SP Narr KL Toga A Hunter AM. Biomarkers to predict antidepressant response. Curr Psychiatry Rep (2010) 12(6):553–62. doi: 10.1007/s11920-010-0160-4 PMC2965366 20963521

[38] Ansar W Ghosh S. Inflammation and inflammatory diseases, markers, and mediators: Role of CRP in some inflammatory diseases. In: Biology of C Reactive Protein in Health and Disease. New Delhi: Springer (2016). doi: 10.1007/978-81-322-2680-2_4

[39] Laudes IJ Guo RF Riedemann NC Speyer C Craig R Sarma JV. Disturbed homeostasis of lung intercellular adhesion molecule-1 and vascular cell adhesion molecule-1 during sepsis. Am J Pathol (2004) 164(4):1435–45. doi: 10.1016/S0002-9440(10)63230-0 PMC1615350 15039231

[40] Amalakuhan B Habib SA Mangat M Reyes LF Rodriguez AH Hinojosa CA. Endothelial adhesion molecules and multiple organ failure in patients with severe sepsis. Cytokine (2016) 88:267–73. doi: 10.1016/j.cyto.2016.08.028 PMC5121929 27701021

[41] Meyer NJ Reilly JP Anderson BJ Palakshappa JA Jones TK Dunn TG. Mortality benefit of recombinant human interleukin-1 receptor antagonist for sepsis varies by initial interleukin-1 receptor antagonist plasma concentration. Crit Care Med (2018) 46(1):21–8. doi: 10.1097/CCM.0000000000002749 PMC5734955 28991823

[42] Casella G Garzetti L Gatta AT Finardi A Maiorino C Ruffini F. IL4 induces IL6-producing M2 macrophages associated to inhibition of neuroinflammation *in vitro* and *in vivo* . J Neuroinflamm (2016) 13(1):139. doi: 10.1186/s12974-016-0596-5 PMC4895901 27266518

[43] Schulte W Bernhagen J Bucala R. Cytokines in sepsis: Potent immunoregulators and potential therapeutic targets - an updated view. Mediators Inflammation (2013) 2013:165974. doi: 10.1155/2013/165974 PMC3703895 23853427

[44] Yang M Meng F Wang K Gao M Lu R Li M. Interleukin 17A as a good predictor of the severity of mycoplasma pneumoniae pneumonia in children. Sci Rep (2017) 7(1):12934. doi: 10.1038/s41598-017-13292-5 29021577 PMC5636901

[45] Morrow KN Coopersmith CM Ford ML. IL-17, IL-27, and IL-33: A novel axis linked to immunological dysfunction during sepsis. Front Immunol (2019) 10:1982. doi: 10.3389/fimmu.2019.01982 31507598 PMC6713916

[46] Noman MZ Desantis G Janji B Hasmim M Karray S Dessen P. PD-L1 is a novel direct target of HIF-1α, and its blockade under hypoxia enhanced: MDSC-mediated T cell activation. J Exp Med (2014) 211(5):781–90. doi: 10.1084/jem.20131916 PMC4010891 24778419

[47] Cao D Hou M Guan YS Jiang M Yang Y Gou HF. Expression of HIF-1alpha and VEGF in colorectal cancer: Association with clinical outcomes and prognostic implications. BMC Cancer (2009) 9:432. doi: 10.1186/1471-2407-9-432 20003271 PMC2797529

[48] Walmsley S Harris A Thompson AAR Whyte MKB. HIF-mediated innate immune responses: cell signaling and therapeutic implications. Hypoxia (2014) 2014(2):47–58. doi: 10.2147/hp.s50269 PMC5045056 27774466

[49] Cai J Wang D Zhang G Guo X. The role of PD-1/PD-L1 axis in treg development and function: Implications for cancer immunotherapy. OncoTarg Ther (2019) 12:8437–45. doi: 10.2147/OTT.S221340 PMC6800566 31686860

[50] Kazanova A Rudd CE. Programmed cell death 1 ligand (PD-L1) on T cells generates treg suppression from memory. PloS Biol (2021) 19(5):e3001272. doi: 10.1371/journal.pbio.3001272 34010274 PMC8168839

[51] Qin W Hu L Zhang X Jiang S Li J Zhang Z. The diverse function of PD-1/PD-L pathway beyond cancer. Front Immunol (2019) 10:2298. doi: 10.3389/fimmu.2019.02298 31636634 PMC6787287

[52] Kim I Moon SO Kim SH Kim HJ Koh YS Koh GY. Vascular endothelial growth factor expression of intercellular adhesion molecule 1 (ICAM-1), vascular cell adhesion molecule 1 (VCAM-1), and e-selectin through nuclear factor-κB activation in endothelial cells. J Biol Chem (2001) 276(10):7614–20. doi: 10.1074/jbc.M009705200 11108718

[53] Carr AC Spencer E Hoskin TS Rosengrave P Kettle AJ Shaw G. Circulating myeloperoxidase is elevated in septic shock and is associated with systemic organ failure and mortality in critically ill patients. Free Radical Biol Med (2020) 152:462–8. doi: 10.1016/j.freeradbiomed.2019.11.004 31698081

[54] Bonaventura A Carbone F Vecchié A Meessen J Ferraris S Beck E. The role of resistin and myeloperoxidase in severe sepsis and septic shock: Results from the ALBIOS trial. Eur J Clin Invest (2020) 50(10):e13333. doi: 10.1111/eci.13333 32585739

[55] Thanabalasuriar A Chiang AJ Morehouse C Camara M Hawkins S Keller AE. PD-L1+ neutrophils contribute to injury-induced infection susceptibility. Sci Adv (2021) 7(10):eabd9436. doi: 10.1126/sciadv.abd9436 33674305 PMC7935370

[56] Wang JF Wang YP Xie J Zhao ZZ Gupta S Guo Y. Upregulated PD-L1 delays human neutrophil apoptosis and promotes lung injury in an experimental mouse model of sepsis. Blood (2021) 138(9):806–10. doi: 10.1182/blood.2020009417 34473230

[57] Obasi T Omair Shafiq M. Towards comparing and using machine learning techniques for detecting and predicting heart attack and diseases, in: Proceedings - 2019 IEEE International Conference on Big Data, Big Data 2019, IEEE International Conference on Big Data (Big Data), Los Angeles, CA, USA, (2019) 2019:2393–402. doi: 10.1109/BigData47090.2019.9005488

[58] Sakar BE Serbes G Sakar CO. Analyzing the effectiveness of vocal features in early telediagnosis of parkinson’s disease. PloS One (2017) 12(8):e0182428. doi: 10.1371/journal.pone.0182428 28792979 PMC5549905

[59] Alfred R Obit JH. The roles of machine learning methods in limiting the spread of deadly diseases: A systematic review. Heliyon (2021) 7(6):e07371. doi: 10.1016/j.heliyon.2021.e07371 34179541 PMC8219638

[60] Kraus VB. Biomarkers as drug development tools: Discovery, validation, qualification and use. Nat Rev Rheumatol (2018) 14(6):354–62. doi: 10.1038/s41584-018-0005-9 29760435

