# Supplementary material for: Model-interpreted outcomes of artificial neural networks classifying immune biomarkers associated with severe infections in ICU
Source: Front Immunol. 2023 Mar 9;14:1137850. doi: 10.3389/fimmu.2023.1137850 (PMC10034398; doi:10.3389/fimmu.2023.1137850)
Supplement: Supplementary file 1 [file DataSheet_1.docx]

Supplementary Material S1

| Biomarker | Function | Reference |
| --- | --- | --- |
| Angiopoietin 2 | Induces vascular sprouting, being upregulated in inflammatory diseases. Higher levels have been associated with prognostics. | Scholz *et* al, 2015  Yang *et* al, 2016 |
| C-C Chemokine Ligand 2 | Its upregulation is associated with higher inflammation as it recruits immune cells to the site of infection. High levels of CCl2 are positively correlated with organ dysfunction. | Gschwandtner *et* al, 2019  Tian *et* al, 2019 |
| C-X-C Motif Chemokine Ligand 10 | High levels of this chemokine were found in critically ill COVID-19 patients. | Gudowska-Sawczuk & Mroczko, 2022 |
| D-dimer | D-dimer is elevated in patients with COVID-19. D-dimer levels correlate with disease severity and are a reliable prognostic marker for in-hospital mortality in patients admitted for COVID-19. | Yao *et* al, 2020 |
| E-selectin | High soluble form of E-selectin was found in the plasma of sepsis patients undergoing critical care. | Cummings *et* al, 1997 |
| Ferritin | Ferritin is associated with the severity of lung involvement for COVID-19 patients. | Knovich *et* al, 2009  Carubbi *et* al, 2021 |
| Granulocyte Colony-Stimulating Factor | No evidences of successful therapies based on the use of G-CSF were found. |  |
| Granulocyte Macrophage Colony-Stimulating Factor | GM-CSF-based therapies have improved recovery from infection, shorter hospital stay, and less days requiring mechanical ventilation. | Mathias *et* al, 2015 |
| Granzyme B | It was found to be an activator of T cells, driving anti-inflammatory responses. | Chung *et* al, 2022 |
| Inter Cellular Adhesion Molecule 1 | A positive correlation between circulating levels of ICAM-1 and septic shock severity was found. | Sessler *et* al, 1995 |
| Interferon ɣ | Immunotherapy based on IFNγ improved immune host defense in sepsis induced immune suppression. | Payen *et* al, 2019 |
| Interleukin 1 β | The IL1 pathway is found highly upregulated in severe COVID-19. | van der Veedonk and Netea, 2020. |
| Interleukin 1 receptor antagonist | Inhibitor of Interleukin 1 β |  |
| Interleukin 2 | It was found elevated in severe patients but decreased in critical COVID patients. | Shi *et* al, 2020 |
| Interleukin 4 | mRNA expression of IL-4 was associated with survival of patients with severe sepsis, but that the plasma IL-4 levels in septic patients on the day of admission to the hospital did not differ between survivors and no survivors | Schulte *et* al, 2013 |
| Interleukin 6 | COVID-19 progression and complications have been associated with higher IL6 serum levels. | Sanli *et* al, 2021 |
| Interleukin 7 | Assists in the survivability and production of immune cells, granting it an anti-viral activity. | Laterre *et* al, 2020 |
| Interleukin 10 | Limits the host immune response | Islam *et* al, 2021 |
| Interleukin 12 | Patients who die as a result of severe sepsis had lower levels of IL-12 produce by PBMCs stimulated by LPS | Wu *et* al, 2011 |
| Interleukin 15 | This cytokine was found to enable the development of septic shock by maintaining the number of Natural Killer cells and their integrity. | Guo *et* al, 2017 |
| Interleukin 17a | Consistently, elevated level of IL-17A is apparently related with disease severity in sepsis. | Ge *et* al, 2020 |
| Lipocalin-2 | Higher regulation of Lipocalin-2 during sepsis indicated it could be a potential marker for the diagnosis of sepsis. | Vasquez *et* al. 2015 |
| Myeloperoxidase | It was shown to be upregulated in critical stages of inflammation in sepsis, granting this biomarker a role of predicting mortality and diagnosing sepsis. | Schrijver *et* al, 2017 |
| Programmed Death-Ligand 1 | It is upregulated on neutrophils during sepsis, which may cause immunosuppression. | Wang *et* al, 2015 |
| Soluble glycoprotein 130 | It is part of the IL6 components. Its higher concentration aids to neutralize the pro-inflammatory effect of IL6:sIL6 complex. | Scheller *et* al, 2011 |
| Soluble interleukin 6 receptor | Soluble form of IL6. |  |
| Surfactant Protein | It is a biomarker that signals lung injury. | Sorensen, 2018 |
| Tumor Necrosis Factor-alpha | Higher concentrations presented a good value in diagnosing sepsis. | Zhai *et* al, 2021 |
| Vascular Cell Adhesion Molecule 1 | High levels of plasma VCAM can be used as a predictive factor of Multi Organ Dysfunction Syndrome is sepsis patients. | Laudes *et* al, 2004 |
| Vascular Endothelial Growth Factor C | It has a prognostic value and can be used as an early diagnostic tool for multi-organ disfunction in sepsis and septic shock. | Almasy *et* al, 2020 |

References

Scholz, A., Plate, K. H., & Reiss, Y. (2015). Angiopoietin-2: A multifaceted cytokine that functions in both angiogenesis and inflammation. *Annals of the New York Academy of Sciences*, *1347*(1). https://doi.org/10.1111/nyas.12726

Gschwandtner, M., Derler, R., & Midwood, K. S. (2019). More Than Just Attractive: How CCL2 Influences Myeloid Cell Behavior Beyond Chemotaxis. In *Frontiers in Immunology* (Vol. 10). https://doi.org/10.3389/fimmu.2019.02759

Gudowska-Sawczuk, M., & Mroczko, B. (2022). What Is Currently Known about the Role of CXCL10 in SARS-CoV-2 Infection? In *International Journal of Molecular Sciences* (Vol. 23, Issue 7). https://doi.org/10.3390/ijms23073673

Yao, Y., Cao, J., Wang, Q., Shi, Q., Liu, K., Luo, Z., Chen, X., Chen, S., Yu, K., Huang, Z., & Hu, B. (2020). D-dimer as a biomarker for disease severity and mortality in COVID-19 patients: A case control study. *Journal of Intensive Care*, *8*(1). https://doi.org/10.1186/s40560-020-00466-z

Cummings, C. J., Sessler, C. N., Beall, L. D., Fisher, B. J., Best, A. M., & Fowler, A. A. (1997). Soluble E-selectin levels in sepsis and critical illness: Correlation with infection and hemodynamic dysfunction. *American Journal of Respiratory and Critical Care Medicine*, *156*(2 I). https://doi.org/10.1164/ajrccm.156.2.9509017

Carubbi, F., Salvati, L., Alunno, A., Maggi, F., Borghi, E., Mariani, R., Mai, F., Paoloni, M., Ferri, C., Desideri, G., Cicogna, S., & Grassi, D. (2021). Ferritin is associated with the severity of lung involvement but not with worse prognosis in patients with COVID-19: data from two Italian COVID-19 units. *Scientific Reports*, *11*(1). https://doi.org/10.1038/s41598-021-83831-8

Mathias, B., Szpila, B. E., Moore, F. A., Efron, P. A., & Moldawer, L. L. (2015). A review of GM-CSF therapy in sepsis. *Medicine (United States)*, *94*(50). https://doi.org/10.1097/MD.0000000000002044

Chung, J. H., Ha, J. S., Choi, J., Kwon, S. M., Yun, M. S., Kim, T., Jeon, D., Yoon, S. H., & Kim, Y. S. (2022). Granzyme B for predicting the durable clinical benefit of anti-PD-1/PD-L1 immunotherapy in patients with non-small cell lung cancer. *Translational Cancer Research*, *11*(2). https://doi.org/10.21037/tcr-21-2506

Sessler, C. N., Windsor, A. C., Schwartz, M., Watson, L., Fisher, B. J., Sugerman, H. J., & Fowler, A. A. (1995). Circulating ICAM-1 is increased in septic shock. *American Journal of Respiratory and Critical Care Medicine*, *151*(5). https://doi.org/10.1164/ajrccm.151.5.7735595

Payen, D., Faivre, V., Miatello, J., Leentjens, J., Brumpt, C., Tissières, P., Dupuis, C., Pickkers, P., & Lukaszewicz, A. C. (2019). Multicentric experience with interferon gamma therapy in sepsis induced immunosuppression. A case series. *BMC Infectious Diseases*, *19*(1). https://doi.org/10.1186/s12879-019-4526-x

van de Veerdonk, F. L., & Netea, M. G. (2020). Blocking IL-1 to prevent respiratory failure in COVID-19. In *Critical Care* (Vol. 24, Issue 1). https://doi.org/10.1186/s13054-020-03166-0

Shi, H., Wang, W., Yin, J., Ouyang, Y., Pang, L., Feng, Y., Qiao, L., Guo, X., Shi, H., Jin, R., & Chen, D. (2020). The inhibition of IL-2/IL-2R gives rise to CD8+ T cell and lymphocyte decrease through JAK1-STAT5 in critical patients with COVID-19 pneumonia. *Cell Death and Disease*, *11*(6). https://doi.org/10.1038/s41419-020-2636-4

Schulte, W., Bernhagen, J., & Bucala, R. (2013). Cytokines in sepsis: potent immunoregulators and potential therapeutic targets—an updated view. *Mediators of inflammation*, 2013.

Sanli, D. E. T., Altundag, A., Kandemirli, S. G., Yildirim, D., Sanli, A. N., Saatci, O., Kirisoglu, C. E., Dikensoy, O., Murrja, E., Yesil, A., Bastan, S., Karsidag, T., Akinci, I. O., Ozkok, S., Yilmaz, E., Tuzuner, F., Kilercik, M., & Ljama, T. (2021). Relationship between disease severity and serum IL-6 levels in COVID-19 anosmia. *American Journal of Otolaryngology - Head and Neck Medicine and Surgery*, *42*(1). https://doi.org/10.1016/j.amjoto.2020.102796

Laterre, P. F., François, B., Collienne, C., Hantson, P., Jeannet, R., Remy, K. E., & Hotchkiss, R. S. (2020). Association of Interleukin 7 Immunotherapy with Lymphocyte Counts among Patients with Severe Coronavirus Disease 2019 (COVID-19). *JAMA Network Open*, *3*(7). https://doi.org/10.1001/jamanetworkopen.2020.16485

Wu, HP., Shih, CC., Lin, CY. et al. Serial increase of IL-12 response and human leukocyte antigen-DR expression in severe sepsis survivors. *Crit Care* 15, R224 (2011). https://doi.org/10.1186/cc10464

Guo, Y., Luan, L., Patil, N. K., Wang, J., Bohannon, J. K., Rabacal, W., Fensterheim, B. A., Hernandez, A., & Sherwood, E. R. (2017). IL-15 Enables Septic Shock by Maintaining NK Cell Integrity and Function. *The Journal of Immunology*, *198*(3). https://doi.org/10.4049/jimmunol.1601486

Ge, Y., Huang, M., & Yao, Y. M. (2020). Biology of Interleukin-17 and Its Pathophysiological Significance in Sepsis. In *Frontiers in Immunology* (Vol. 11). https://doi.org/10.3389/fimmu.2020.01558

Vazquez, D. E., Niño, D. F., de Maio, A., & Cauvi, D. M. (2015). Sustained expression of lipocalin-2 during polymicrobial sepsis. *Innate Immunity*, *21*(5). https://doi.org/10.1177/1753425914548491

Schrijver, I. T., Kemperman, H., Roest, M., Kesecioglu, J., & de Lange, D. W. (2017). Myeloperoxidase can differentiate between sepsis and non-infectious SIRS and predicts mortality in intensive care patients with SIRS. *Intensive Care Medicine Experimental* , *5*(1). https://doi.org/10.1186/s40635-017-0157-y

Wang, J.-F., Li, J.-B., Zhao, Y.-J., Yi, W.-J., Bian, J.-J., Wan, X.-J., Zhu, K.-M., & Deng, X.-M. (2015). Up-regulation of Programmed Cell Death 1 Ligand 1 on Neutrophils May Be Involved in Sepsis-induced Immunosuppression. *Anesthesiology*, *122*(4). https://doi.org/10.1097/aln.0000000000000525

Scheller, J., Chalaris, A., Schmidt-Arras, D., & Rose-John, S. (2011). The pro- and anti-inflammatory properties of the cytokine interleukin-6. In *Biochimica et Biophysica Acta - Molecular Cell Research* (Vol. 1813, Issue 5). https://doi.org/10.1016/j.bbamcr.2011.01.034

Sorensen, G. L. (2018). Surfactant protein D in respiratory and non-respiratory diseases. In *Frontiers in Medicine* (Vol. 5, Issue FEB). https://doi.org/10.3389/fmed.2018.00018

Zhai, G. H., Zhang, W., Xiang, Z., He, L. Z., Wang, W. W., Wu, J., & Shang, A. Q. (2021). Diagnostic Value of sIL-2R, TNF-α and PCT for Sepsis Infection in Patients With Closed Abdominal Injury Complicated With Severe Multiple Abdominal Injuries. *Frontiers in Immunology*, *12*. https://doi.org/10.3389/fimmu.2021.741268

Laudes, I. J., Guo, R. F., Riedemann, N. C., Speyer, C., Craig, R., Sarma, J. V., & Ward, P. A. (2004). Disturbed homeostasis of lung intercellular adhesion molecule-1 and vascular cell adhesion molecule-1 during sepsis. *The American journal of pathology*, 164(4), 1435–1445. https://doi.org/10.1016/S0002-9440(10)63230-0

Almasy, E., Szederjesi, J., Grigorescu, B. L., Badea, I., Petrisor, M., Manasturean, C., Negrea, V., Timar, A.-E., Coman, O., Azamfirei, L., Santini, A., & Copotoiu, S. M. (2020). The Diagnostic and Prognostic Role of Vascular Endothelial Growth Factor C in Sepsis and Septic Shock. *The Journal of Critical Care Medicine*, *6*(3). https://doi.org/10.2478/jccm-2020-0020
