# Supplementary material for: Model-interpreted outcomes of artificial neural networks classifying immune biomarkers associated with severe infections in ICU
Source: Front Immunol. 2023 Mar 9;14:1137850. doi: 10.3389/fimmu.2023.1137850 (PMC10034398; doi:10.3389/fimmu.2023.1137850)
Supplement: Supplementary file 3 [file DataSheet_3.pdf]

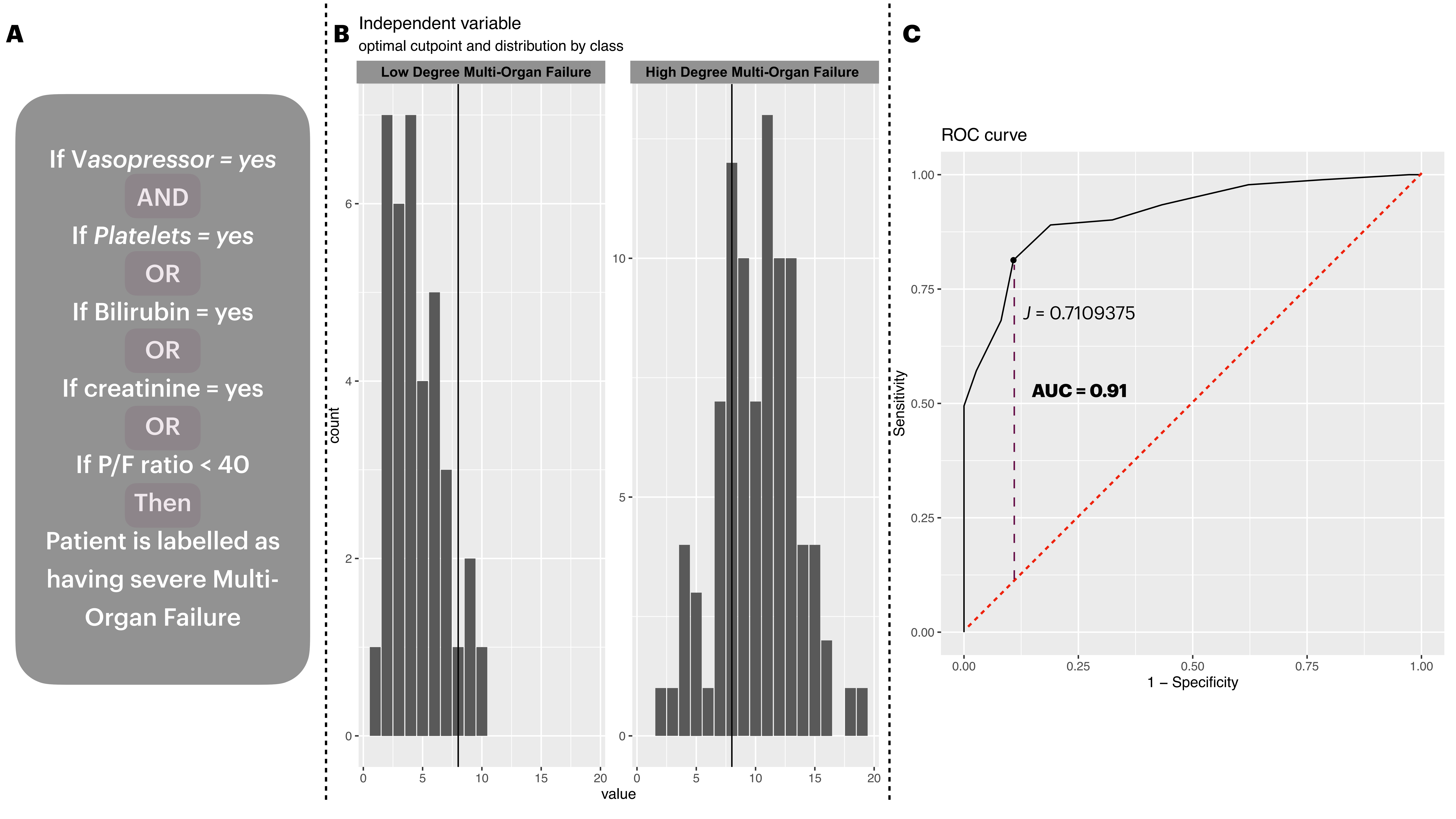
A
If Vasopressor = yes
AND
If Platelets = yes
OR
If Bilirubin = yes
OR
If creatinine = yes
OR
If P/F ratio < 40
Then
Patient is labelled as having severe Multi-Organ Failure
B
Independent variable
optimal cutpoint and distribution by class
Low Degree Multi-Organ Failure
High Degree Multi-Organ Failure
count
value
C
ROC curve
J = 0.7109375
AUC = 0.91
Sensitivity
1 – Specificity
